# Areas for Improvement and Barriers Identified in Measuring the Quality of Nursing Care: Perceptions of Patients and Professionals

**DOI:** 10.3390/nursrep14040211

**Published:** 2024-10-09

**Authors:** Maria Consuelo Company-Sancho, Manuel Rich-Ruiz, Marta Guillen Toledano, Luis-Miguel Cairós-Ventura, Josefa D. Gil Perez, Ana María de Pascual y Medina, María Pilar Peláez Alba, Ana Isabel Barreno Estévez, María Emma Alonso Poncelas, Carolina Rodríguez Orihuela, Pedro Ruyman Brito-Brito

**Affiliations:** 1 Health Promotion Service, Directorate-General for Public Health, Canary Islands Health Service, 35003 Las Palmas de Gran Canaria, Spain; chelocomp@gmail.com; 2 Nursing and Healthcare Research Unit (Investén-Isciii), Instituto de Salud Carlos III, 28029 Madrid, Spain; 3 Research Network on Chronicity Primary Care and Prevention and Health Promotion (RICAPPS), 38109 Canarias, Spain; 4 Instituto Maimónides de Investigación Biomédica de Córdoba (IMIBIC), Universidad de Córdoba (UCO), Hospital Universitario Reina Sofía (HURS), 14004 Cordoba, Spain; 5 CIBER on Frailty and Healthy Ageing (CIBERFES), Instituto de Salud Carlos III, 28029 Madrid, Spain; 6 Directorate-General for Patients and Chronicity, Canary Islands Health Service, 35004 Las Palmas de Gran Canaria, Spain; mguitol@gobiernodecanarias.org; 7 Santa Cruz de la Palma Health Centre, Canary Islands Health Service, 38700 La Palma, Spain; lcaiven@gobiernodecanarias.org; 8 Nursing Department, Faculty of Healthcare Sciences, University of la Laguna, 38200 Santa Cruz de Tenerife, Spain; pbritobr@ull.edu.es; 9 Puerto del Rosario Health Center, Canary Islands Health Service, 35600 Fuerteventura, Spain; josefadolores.gil@ulpgc.es; 10 Evaluation of the Canary Islands Health Service (SESCS), 38109 Tenerife, Spain; anamaria.pascualmedina@sescs.es; 11 Directorate-General for Care Programs, Canary Islands Health Service, 38071 Santa Cruz de Tenerife, Spain; mpelalb@gobiernodecanarias.org; 12 Epidemiology and Surveillance and Prevention Service, Directorate-General for Public Health, Canary Islands Health Service, 35003 Las Palmas de Gran Canaria, Spain; ana@laenfermera.es; 13 Hospital Doctor José Molina Orosa, Canary Islands Health Service, 35500 Arrecife, Spain; ealopon@gobiernodecanarias.org; 14 Complejo Hospitalario Nuestra Señora de Candelaria, Canary Islands Health Service, 38010 Santa Cruz de Tenerife, Spain; crodori@gobiernodecanarias.org

**Keywords:** quality indicators, nursing care, patient satisfaction, qualitative research

## Abstract

Background: Quality indicators in healthcare are essential to raising awareness about the appropriateness of nursing care. However, identifying the key indicators continues to pose a challenge, above all if one wants to include users and professionals. Aim: Identify areas for improvement and potential barriers to measuring the quality of nursing care in a regional health service. Methodology: Interpretive qualitative exploratory study. The participants were users and professionals of an autonomous regional health service in Spain. The information was collected by means of two focus groups and eight semi-structured interviews conducted between November 2022 and March 2024. The data were analysed thematically using the Braun and Clarke process. Results: The users and professionals agreed on the need to measure emotional skills and attitudes such as empathy, respect, and warmth when dealing with patients. The professionals highlighted workload and inefficiency of the current record system as barriers to adequately reflecting their work. They proposed improvements in the recording tool and the need for more human resources, as well as leadership that is more focused on quality. Conclusions: It is crucial to develop indicators that reflect both the emotional and technical aspects of nursing care. The participation by patients and professionals alike in this design and selection will make it possible to improve the quality of care and advances in the nursing profession. This study was not registered.

## 1. Introduction

Indicators are valid and reliable metrics related to performance [1]. They are specific markers that make quality visible to healthcare stakeholders. The use of appropriate and pertinent indicators sensitive to nursing activity provides the opportunity to demonstrate the contributions of these professionals to the quality results of the care offered to users [2]. Their evaluation enables the advancement of the nursing profession, the definition of professional functions in their various dimensions, the standardisation and recording of diagnoses, objectives, and interventions, the adequacy of the nurse/patient ratio, the management of organisations, and research in the discipline [3].

Nurses have come a long way in the development of competence-based frameworks for the provision of high-quality care [4]. However, the identification of key performance indicators and the definition of the results expressed by users, who are directly or indirectly affected as the work provided by nurses is not easily assessed, is still a matter of debate [5] (chapter 5). There is significant heterogeneity in the definition and interpretation of indicators, with contextual differences further complicating standardisations [6].

In addition, care can be intangible, dynamic, context-dependent, and heterogeneous, so establishing indicators presents a significant challenge and requires the participation of the parties involved: professionals, patients, and their families. Factors that influence quality of care can include environment and culture, training and skills, infrastructure, and organisation [7], and are key for developing sensitive indicators.

In the literature on nursing indicators, topics such as patient safety, compliance with protocols and clinical practice guidelines, and rates of adverse effects, among others, stand out. These indicators are essential for assessing the quality of care, but they can be improved by incorporating the perspectives of patients and professionals, helping to identify and prioritise the issues that really matter, and promoting more personalised and effective care [8].

Nurses should be involved in the design and selection of quality indicators to ensure that they adequately reflect the work and contribution of the profession to patient care [9]. The reality experienced in clinical practice, taking into account aspects of the organisational culture and environment in which the indicator will be measured, will help to complement the theoretical construction of the indicators [10]. Implementing participatory evaluation research that explores both the structure and the process of the quality of the indicators, with the participation of all the actors involved, encourages knowledge transfer, the transformation of practices, and organisational change in general [11]. While nurses are the appropriate informants to suggest, based on reflective practice, quality indicators suited to their local circumstances [12], in most of the studies reviewed there is a lack of formal discussion from professionals and active participation in their definition and application.

Incorporating patient perspectives into the process of drug development, health education, service delivery, and research is increasingly recognised, providing better outcomes and benefits for all stakeholders [13]. The benefit of engagement by the consumers of these resources allows for influencing the health agenda from the development of services to the identification of research priorities, providing greater ownership of systems [14]. In health research, its incorporation is part of a family of participatory research methods that aim to transfer power from the researcher to the research participant [15]. There are studies that demonstrate the need for patients to participate in decision making regarding nursing care [16]. Much of the research incorporates users once the indicator is already developed, as these are required for evaluation [17].

An analysis through qualitative techniques makes it possible to describe the practices under transformation and the development of theoretical foundations, giving rise to well-founded knowledge about lived realities [11]. The focus group, as one of these techniques, allows for dialogue on an issue lived and shared through common experiences [18]. Participants can maintain their initial opinions, change them, or adopt new ideas based on the reflections discussed in the group [19]. The individual interview allows the researcher to unravel meanings developed by the subjects through discourses, stories, and experiences, addressing the subject in their intimacy and individuality [20].

Taking into account the need to create valid and reliable indicators with the actors involved, the main objective of this study is to identify areas for improvement and potential barriers to measuring the quality of nursing care in a regional health service.

## 2. Materials and Methods

### 2.1. Design

This is qualitative, exploratory, and interpretative research, in which the information obtained from the participants on the contents and way in which nursing care should be measured has been combined with the researchers’ understanding of the situation [21].

The research is part of a project funded by the Canary Islands Health Research Institute Foundation (FIISC), file ENF21/12 in its 2021 call, which included a first phase of systematic peer review of the literature, a second qualitative exploratory phase (included in this study), and a third phase in which, using the Delphi method, a prioritisation of the indicators proposed in the first and second phases was carried out.

### 2.2. Scope

The study was carried out in the context of a regional health service, the Canary Health Service (SCS) of the Canary Islands, within the Spanish National Health System, which serves a population of two million inhabitants. This territory is made up of seven health areas, with their reference hospitals and primary care centres. All areas and scopes participated.

### 2.3. Participants

The participants were both users and professionals. The professionals were intentionally selected by a gatekeeper (located in the centre) from different care settings (specialised and primary care), and different centres, according to their knowledge, their ability, and their willingness to talk about the subject. They were of different ages and their professional experience differed. Users were also selected intentionally by a medical or nursing professional from the health centre or hospital known to them, who invited them to participate, as well as through patients’ associations. Users were also selected with a variety of situations such as age, the processes/diseases for which they required care, and place of residence (both rural and urban areas). 

The final number of informants was determined by the criterion of discourse saturation. That is, information collection continued up to the point at which the research team assessed that the necessary elements to build up a comprehensive and convincing framework of the topic of interest were identified [22], in our case, the development of a set of indicators for the quality of clinical nursing practice that can be entered, a posteriori, into the health history of the SCS.

A total of 28 participants were recruited; among them, 20 participated in the focus groups (10 in the professional group, 10 in the patient group), and 8 participated in the individual interviews.

### 2.4. Data Collection

Two focus groups and eight semi-structured interviews were conducted between November 2022 and March 2024. One of the focus groups was carried out with professionals and the other with SCS users. All the members of the professional focus group were SCS workers. Professionals from the private health sector were not included in the study. All participants were asked to sign an informed consent form. 

Prior to the group and individual interviews, a first draft script was prepared in accordance with the starting categories (Appendix A). All the individual interviews were conducted online via the Webex® platform (https://www.webex.com/, accessed on 30 November 2022). As for the groups, the patient group was interviewed in person while the professional group was interviewed online via the Webex® platform. When conducting the interviews, an attempt was made to create a climate conducive to communication. The initial questions were asked in an open-ended, non-leading manner so that the interviewees could answer freely. In the case of the focus groups, in addition to the moderator, an external observer took notes faithfully on everything that was said in the group. All interviews were audio-recorded and subsequently transcribed in full in accordance with a transcription script.

As for the organisation of the professional focus group, the following heterogeneity criteria were followed: (1) age and work experience, 20–30 years old (<10 years of work experience), 30–50 years old (10–25 years of work experience), and >50 years old (more than 25 years of work experience); (2) professional practice in primary care, in urban or rural health centres, local clinics, and out-patient emergencies; and (3) professional practice in hospital care, in hospitalisation units (chronic illnesses and long admissions), surgical hospitalisation (short illnesses, in general), mother and child hospitalisation, and hospitalisation in special services (intensive care/emergency/surgical ward).

The heterogeneity criteria in the focus group of users were as follows: (1) residents of different islands and areas, (2) those who have had some contact as a user of the Canary Islands Health Service, (3) those who belong to primary care and AE, and (4) wanting to participate in the study.

To ensure the anonymisation and confidentiality of the participants, their names were coded (from the transcripts onward) as follows: (a) the acronym “E-PROF” (professional) followed by an assigned number or “E-PAT” (patient), followed by a number, would be used, based on the order of the individual interviews conducted; (b) in the case of the participants interviewed in groups, they were coded as “G-PROF” (professional group) plus the number assigned within the group, and “G-PAT” (patient group), again followed by a number, to identify the participants in the focus groups.

### 2.5. Data Analysis

A thematic analysis was carried out following the process defined by Clarke and Braun, [23], to capture a set of themes representative of the content collected. To achieve this, the following phases were followed: (1) familiarisation with the data; (2) generation of the first codes; (3) search for topics; (4) review of the topics; (5) definition and titling of the topics; and (6) preparation of the results report.

### 2.6. Rigour

The rigour and quality criteria followed in this research were those established by Calderón [24], that is, (a) theoretical, epistemological, and methodological adequacy: thus, the discourse of users and professionals, combined with the understanding of the researchers, will allow the appropriate identification of aspects/variables that can be measured by other techniques in subsequent studies; (b) relevance: knowledge of the aspects of nursing care that professionals and users believe should be measured and how they should be measured will help to identify a system for evaluating the quality of nursing care in hospital care and primary care in the Canary Islands Health Service; (c) validity: a triangulation was carried out between techniques and among the research team. In addition, during the analysis, each defined code/category was constantly cross-checked with the original transcripts; and (d) reflexivity: the authors feel especially motivated and sensitised to nursing care. Several researchers work as technical staff in several bodies or services that are part of the SCS. This emic perspective required, a priori, the sharing among the research team of visions and beliefs related to the subject, so that possible biases that could have arisen from this circumstance were eliminated.

### 2.7. Ethical Considerations

The ethical principles of the Declaration of Helsinki and the standards of Good Clinical Practice in research were respected. The study was approved on 27 August 2021 by the clinical research ethics committee of the Dr. Negrin University Hospital, Gran Canaria (Code: 2021-336-1). The study complies with the provisions of Organic Law 3/2018, of 5 December, on the Protection of Personal Data and Guarantee of Digital Rights, as well as Regulation (EU) 2016/679 of the European Parliament and of the Council of 27 April 2016 on Data Protection.

## 3. Results

The results of the study reflect the different (and at the same time, complementary) views with which users and professionals face the question of which aspects of nursing care should be measured and how. While the former focus their attention on qualities that they value (positively) in nurses, the latter focus on instrumental and organisational aspects that hinder obtaining more and better data in this regard. In any case, both users and professionals agree on highlighting the emotional work of nurses and the difficulties that staff shortages and, consequently, lack of time and the resulting instability, have on the care provided.

For a detailed description of the findings of the study, the following sections were created: (1) what should be considered when measuring a nurse’s work; (2) what is the baseline situation; (3) possible solutions; and (4) advantages of measuring nursing work.

### 3.1. What Should Be Taken into Account to Measure a Nurse’s Work

With regard to one of the fundamental objectives of this study, namely, which aspects of nursing care should be measured, users and professionals agree in pointing out the entire repertoire of skills that have to do with the emotional work of nurses, such as not taking things for granted and knowing how to listen, not judging, having empathy and knowing how to understand, compassion [encouraging and supporting], and “getting you out of the situation you’re in”.

“For me, active listening is essential, to be listened to, sitting down and telling them how you are, how you feel, what concerns you have, […]”.

(G-PAC 1)

“I want a person who can guide me, and who puts themself in my shoes; [but] that doesn’t judge me, putting yourself in my shoes doesn’t give you the right to tell me that I did things wrong, they can guide me and help me to understand things, but not judge me or lecture me”.

(E-PAT 1)

“[…] My mother was hospitalised, but they wouldn’t let me visit her. It was because of COVID, I called every day, but they wouldn’t let me talk to her and she’s an elderly person. Imagine, I take care of her every day, but they wouldn’t let me visit her. One day, a nurse picked up the phone, I spoke with her and she was very kind. Later she arranged a video call with me on her mobile phone and I was able to talk to my mother. That nurse understood me and put herself in my shoes […]”.

(E-PAC 2)

“I’m a cry-baby, and I know I can cry calmly with him [the nurse] because he’s going to support me and cheer me up and I know that the words he says to me aren’t the typical words that anyone you don’t know might say, you know?”.

(G-PAT 2)

In addition, users believe that attitudes that they identify with the nursing profession should be evaluated. These attitudes include vocation, involvement, respect, tact, warmth, and approachability.

“It’s my belief that to work as a nurse you must have a vocation. It’s something you notice straight away. They live it, that engagement with the patient”.

(E-PAT 3)

“Ultimately, I think it’s a vocation, but apart from a vocation it’s kindness towards the patient; we go through enough already day after day without arriving for a consultation and being treated like “come on, next”, as if you’re just a number […] you need a little kindness from the person who’s taking care of you”.

(G-PAT 3)

“[speaking from personal experience] It was how they treated you. First of all, “so how are you?”, how they welcomed you, how they approached you and the tact with which they did it, […] for me it was [unintelligible], but it was the approach and the warmth”.

(G-PAT 4)

Another important quality, implicit in the users’ discourse, is professionalism, that they [the staff] know and do their job well.

“Technical training and professional expertise are also important”.

(E-PAT 1)

“You notice the authority that knowledge gives you, the person who takes their job seriously, who jokes with you or whatever, but you notice that, at the same time, they have that authority that comes from knowledge”.

(G-PAT 2)

Finally, users believe that nurses’ concern for training should be valued, that “they have that curiosity to continue growing”.

“I believe that the nursing professional should be a professional who is specialised as much as possible, and who has that curiosity to continue growing as a person”.

(G-PAT 5)

### 3.2. Baseline Situation

The professionals agree that there is currently not enough data collected on the contribution of nurses to the care provided by health services, especially when it is “on demand” or in emergencies.

“[…] there is a very big gap between what one actually does and what is recorded […] It’s true that there are nursing actions that we’re doing continually, and often without realising it, and they’re not categorised, they’re not quantified, they’re not written down anywhere”.

(G-PROF 1)

“We work to a schedule, where the set times are usually correct for the activity to be carried out, but, when it is “on demand” or in the Emergency Department, things change because the times do not make allowance for it”.

(E-PROF 1)

Among the reasons that lead to this insufficiency are, fundamentally, the workload. The care workload is so heavy that there is no time to reflect on everything that has been performed in the working day. In addition, the recording itself, through the computer system, also “steals” their time.

“Because you do more in your working day than you can record later in the system, in those 30 min; and very often you leave things unrecorded due to lack of time, because the patient comes first […] and afterward the computer system that also takes up time”.

(G-PROF 2)

Beyond time, professionals believe that the tool currently used to record their work (Electronic Health Record) does not truly reflect everything they do in a working day, that it is designed to enter, above all, quantitative information, and that there is an under-recording of qualitative data.

“Nursing staff do so much that isn’t measurable in terms of objectives and no, its’ not measured, […] Above all, the emotional aspect is not reflected at all, I think, rather it refers more to indicators, patterns, all that, but the emotional aspect isn’t reflected at all”.

(G-PROF 3)

As such, data related to something as intangible as empathy, for example, or the bond that a nurse creates with patients, are not collected.

“[…] I may spend 10 min talking to the family, reassuring the family, but that is not recorded […], everything else is recorded, all the techniques, but as for communication, nothing”.

(G-PROF 4)

In addition, the lack of job stability (perceived and criticised even by users) makes it difficult to maintain these emotional bonds in the professional-patient relationship.

“You have the contact with the person (nursing staff), the trained person, everything’s wonderful, and suddenly, there’s a change of nurses and you are like “well, its’ no big deal”, change…, now the second one”.

(G-PAT 4)

In the opinion of some participants, the prevalence of the quantitative nature when recording the work carried out is related to the purpose of the data, which is mainly aimed at measuring efficiency (in numerical terms), with economic pay-off as the goal.

“In the hospital it is very difficult for the qualitative part to be valued, procedure speed is rewarded and how many people you are able to attend to in a ward, quality is not valued […]”

(E-PROF 2)

“And as for what they were talking about before about the indicators, when they taught me this business about recording, and all this about incentives, sorry, they told me “you have to do this so that you earn more”. I mean, I don’t know, they view it in the end in economic terms, you know? They don’t see it as something that’s good for the patient, which I forgot to say before, I mean, when they taught it to me, they taught it that way”.

(G-PROF 5)

Moreover, professionals also believe that a lot of time and effort is invested into other tasks (“hidden work”) that in the end are not recorded anywhere. They refer, on the one hand, to administrative tasks carried out to “resolve red tape for patients” in an attempt to “address everything they ask of us”, and, on the other hand, to that significant number of tasks or actions carried out by nursing staff and which, initially, have nothing to do with a nurse’s job description.

“The patient comes away with their appointment, although it means more work for the nurse, but it means more quality for the patient. It is regarded as part of the job because of the benefit obtained, “that is to say Quality” for the patient”

(E-PROF 1)

“How much time and energy do we invest in the tasks of other professionals, that other colleagues are supposed to do and that we take on and that, what’s more, since they’re not recorded anywhere, don’t appear anywhere”

(G-PROF 3)

“You move an oxygen cylinder, you take delivery of vaccines or the order from the warehouse or the order from the pharmacy […] The administrative staff are not going to take delivery of it!”

(G-PROF 5)

### 3.3. Solutions and Changes

The solutions pointed out by professionals to combat these limitations point in different directions. However, the most fundamental is that the tool should allow “care to be reflected in a practical way when carrying out and viewing how the patient is evolving” (E-PROF 3).

Along with this demand, another common one is that sufficient time is needed to be able to provide good quality of care:

“We need more time to better listen to patients and examine them with the tools available to us”.

(E-PROF 1)

“[…] There is no other solution, we can do acrobatics, but the time is what it is and people have the time they have”.

(G-PROF 3)

Regarding “hidden work”, they do not propose actions aimed at recording it, but eliminating it. To this end, they identify two basic measures: allocating more human resources and promoting team spirit.

“I think that increasing staff numbers would mean we wouldn’t have to do the work of other staff, right? If there were more staff, obviously you wouldn’t have to do, as colleagues say, things that are not part of your job description”.

(G-PROF 8)

The perception is that people are not being given the care they need. Neither managers nor the colleagues themselves do so. That is why they feel it is crucial to have a team that is supportive and cohesive.

“[They look forward to those moments when it appears] that shared feeling of ‘come on, let’s all pull together, let’s take the [gas] cylinder, we do it and so on’”.

(G-PROF 7)

In addition, the professionals highlight the influence that managers have on their performance and, therefore, on the quality provided by nurses.

Another type of leadership is called for that does not manage shifts, incentives, etc., and “that is not only concerned with the work in quantitative data, but also cares about the quality of the work performed” (E-PROF 1), “[…] that pushes me, that encourages me to write the histories, because that is going to have an impact, not financially with incentives, but of recognition of the profession, that it is measured in some way, clearly, that they acknowledge my good work, is what I feel is lacking in managers” (G-PROF 6).

Speaking of managers, some participants say that “managers know who does more and who does less”, but that, in the end, they do not differentiate.

“In terms of managing the work we do on a day-to-day basis; I think our managers have a lot to do with it, they know who does more, they know who does less. The problem is there’s no differentiation”.

(G-PROF 2)

Lastly, the professionals deemed it important to have the role of a quality manager in hospitals and primary care centres (CAP according to the Spanish acronym).

“I agree with the colleague who was speaking now, who spoke about quality managers. There are many, many hospitals, many health [centres] that are adhering to the idea of quality seals that, in some way, have a format that ensures traceability from the moment the patient enters until the patient leaves the doctor’s surgery or the centre, giving us those patients’ responses to the quality that the patient has received”.

(G-PROF 4)

### 3.4. Advantages of Measuring Nursing Work

In the analysis of the professionals’ data, and of some users, two benefits stand out: (1) the visibility of care; and (2) the possibility of comparison with one another and with other professionals, and of being able to motivate themselves.

“It has been a crucial role (that of the nurse), at least in Primary Care. I didn’t know half of the things they do, and they do a lot of background work, just as doctors do. So, I believe that it should be evaluated to improve and above all bring awareness to all the competencies and functions that nurses can carry out”.

(G-PAT 4)

“By evaluating nurses, they can surely improve, because they can see where they are making mistakes and what things they do well. In addition, they can also see if colleagues are better or not and if they have changed from one year to the next. I think this can motivate them to be better and to develop professionally better, especially technically, which I think is very important”.

(E-PAT 4)

“Assessment is an incentive for a nurse”.

(E-PAC 3)

Lastly, when the professionals were presented with the challenge of summarising, in one or two concepts, the work and contribution of the nursing profession to patients and their health outcomes, they mostly agreed on highlighting patient satisfaction.

“Personally, what reflects my work is the satisfaction of the patient I have treated when they leave…”

(G-PROF 9)

“If the patient is satisfied, the professional is also satisfied”.

(E-PROF 1)

## 4. Discussion

The results show that there is a dual and complementary perspective on the measurement of care between users and professionals. For users, emotional and relational qualities are the most highly valued. For professionals, instrumental and organisational aspects, which make it difficult to gather information on the work they do, are the main difficulties they encounter.

Patient satisfaction increases when nurses demonstrate competence in technical care, skills, and knowledge. This is consistent with other studies in which patients value quality technical care provided by nurses, especially if it is accompanied by a sympathetic attitude and good interpersonal skills [25]. Attitudes such as vocation, respect, and tact are emphasised by the users in the study, with respect, that is, respect for life, dignity, and human rights, inherent to the nursing profession and deontological obligation [26]. Attention to the provision of instrumental care, answering patient needs, respect, assuaging patient anxiety, provision of emotional and qualified support, relief of comfort, and being there for the patient when they need it are some of the results obtained in other studies [27].

These emotional and relational skills, such as empathy, ability to listen, a non-judgemental attitude, and compassion, are also highlighted by professionals when they are measured. A study pointed out that the four ethical values that nurses most highlighted in their care work were as follows: prudence when faced with a critical diagnosis, honesty with the institution in which they work, reliability in the activities that are within their competence, and discretion and kindness [28]. We recommend comparing and contrasting these perceptions of ethical values among family members, patients, and other professionals, for the sake of accuracy in the indicator to be measured. 

Professionalism and the need for continuous training are two other points emphasised by professionals. The use of nursing diagnoses, registration of care plans, and continuing education programmes stand out to maintain the quality of care [29]. It is important to transfer this improvement in continuous training into efficiency, quality, and sustainability indicators [30].

Another problem detected is the lack of data collected on the actions carried out by nurses, especially in on-demand care and emergency services. Records guarantee the quality and continuity of care, improve communication and prevent errors, and, if they are properly filled in, guarantee continuity of care [31]. The professionals argue that the heavy workload, as well as the design of current records, lead to a bias in the recording of quantitative rather than qualitative data. They also advocate for leadership that values both the quality and quantity of the work carried out and the inclusion of quality managers in hospitals and primary care centres; as Zabalegui comments, nursing leadership must also be applied in management activities [32]. Workloads together with the scarcity of resources are among the most sensitive areas in the nursing practice environment. Without sufficient resources, it becomes more difficult for nurses to provide excellent care [33]. Job instability is another of the verbalised findings that negatively affects the continuity of care and can negatively influence the quality level, with a clear relationship between patient safety and nurses’ well-being with a shortage of nursing staff [34].

Nurses are generally considered to multitask and complete nursing care activities in short periods [35]. Our results make visible the amount of work, specifically “hidden work”, that nurses do, which is not measured and is often not part of their job description. In addition, it is suggested that teamwork can alleviate part of this work. Collaboration across professional categories is key to optimising patient outcomes and reducing healthcare costs [36]. In addition, these collaborative dynamics contribute to increasing job satisfaction and maintaining patient safety, which are critical aspects for the quality of care provided [37]. 

There is a causality that links aspects related to the environment in which nurses provide their care with mortality risks [38]). “Missed nursing care” is also important to some extent, both in the literature and in veiled comments in patient interviews [39]. This may help to understand the proven association between fewer nursing professionals and more adverse outcomes with care, highlighting the interconnection and importance of patient safety in nursing interventions. 

Both users and professionals point out that the visibility of nursing work enables better self-assessment and increased motivation to achieve improvements, influencing satisfaction of both patients and nursing professionals. Adequate visibility can positively influence patient satisfaction [40]. 

Further qualitative studies on the perceived quality of care, both by users and professionals, will provide better knowledge of the real situation from the perspective of those experiencing it, a conclusion reached by other studies [41].

### 4.1. Limitations

A limitation of the study lies in the restriction of the sample to public sector professionals, which may have biased the results by not including the perspective of private sector professionals. 

Despite efforts to homogenise the sample in terms of age, gender, and work area, it is likely that there are subgroups of professionals who were not adequately represented. 

Additionally, the selection of users may have favoured those who were more proactive and had a greater interest in participating, which may not reflect the opinion of the general user population.

### 4.2. Implications for Practice

From a management point of view, there is evidence of the value of the opinions of nurses who provide direct care to patients to improve patient satisfaction. 

The development and innovation of tools that incorporate holistic measurements and collect qualitative data can reveal the nurse’s hidden work and the patient’s emotional experience.

Encouraging continuous feedback systems on the quality of care received and delivered can facilitate rapid and personalised adjustments to patient care.

## 5. Conclusions

Users and professionals agree on highlighting nurses’ emotional and relational skills, although users also emphasise the technical competence and the concern of nurses to update their knowledge as important aspects of nursing care that should be measured.

Professionals, on the other hand, focus their feedback on the current challenges of properly recording their activity, rather than on its content. They consider that the computer tool is markedly quantitative and that the focus/purpose given to the recording of the data is largely concerned with efficiency. They add to this the significant workload, lack of resources—also identified by users—a lack of training, and job instability as constraints on their activity. 

The organisational dynamics and tools available in the system seem to influence how professionals provide care, which can make it difficult for nurses to provide accurate and detailed information to patients about their health process, thus compromising their satisfaction and experience of the care received.

The inclusion of both patient and professional points of view in the development of care quality indicators can improve their development.

## Data Availability

Data are contained within the article.

## Public Involvement Statement

There was no public involvement in any aspect of this research.

## Guidelines and Standards Statement

The research followed established qualitative methodologies, specifically utilizing the thematic analysis process outlined by Braun and Clarke. Additionally, the study adhered to the Consolidated Criteria for Reporting Qualitative Research (COREQ) guidelines. We employed strategies such as member checking, triangulation, and detailed documentation to ensure credibility, transferability, dependability, and confirmability of the data. The data analysis was conducted systematically, ensuring transparency and reliability throughout the process.

## Appendix A

Script for Focus Group and Interviews
Introduction

The moderator starts by introducing him or herself, then the participant go around and do the same. It is important to request authorisation to record the session, informing the subjects that the recording will be used solely to transcribe the session, so the analysis will be anonymous. It is also the time to remind them of the focus group rules: respect the turn-taking, there are no right or wrong answers, and what matter is what is said, not who says it.

Description of the study

Nursing care is fundamental in all aspects of patient care, including care techniques, education, treatment, promotion, prevention, support and monitoring, and although measuring care can be complex due to its dynamic and varied nature, monitoring these activities will bolster nurses’ involvement and leadership in the health system.

We understand that all of you have had experiences with the Canary Islands Health Service, either as professionals or as patients. From the nurses’ perspective, it is essential to understand, from their point of view and based on their daily activities, what aspects that should be measured to adequately reflect nursing care. This impacts visibility, quality, and resource allocation, as inclusion in recording systems makes it possible to improve performance, eliminate ineffective practices, and quantify in terms of costs. From the patients’ point of view, we would like to focus on their experience of the care process with the nursing professionals, in any of its forms: attending a nursing consultation, taking blood pressure or administering medication, during hospital admission, etc., and what was most important in those interactions.

Our aim is to improve the quality of care provided by nurses, identifying the aspects and/or components of their professional practice that provide the most value, i.e., what behaviours, attitudes and/or actions patients and nursing professionals consider important, necessary and valuable. Your input will help us create indicators that measure and improve nursing performance in the SCS.

Implementation

Focus group: Patients.

Ask participants to focus on a specific situation in their experience with the health system, focusing on the care received from nurses. Within this context, we ask:

- Regarding the care received from the nurse, what was most important to you?
- What did you like the most (did you find most appropriate)? And what did you like the least?
- Was there anything you found lacking in their performance?

And in a general context, ask:

- Do you think that all nurses should be measured equally? (Intensive care nurses, nurses in a hospitalisation ward, a health centre, etc.)
- What would you highlight as most important and essential in nursing care in the situations we just described?

Focus group: Nursing professionals.

- What is the most important thing about your work as a nurse for you as a professional, for the patients you care for and for your hierarchical superiors?
- Of all the tasks you perform, which do you think can be measured and which should be quantified?
- What aspects of your activities could demonstrate the efficacy, quality, and safety of the care you provide?
- What would an indicator of the care you provide focus on: the results (how much is done) or the process (how it is done)?
- Which aspects do you think should be measured by the institution where you work and in terms of professional incentives?

Individual interview: Patients.

Let’s talk about which aspects of nursing care should be measured.

- [To begin with, could you tell us what a nurse does, what are their roles and responsibilities?]

[You may find the question difficult] If the work of a nurse has to be measured, what do you think should be taken into account? [If they find this difficult, ask specifically:] What do you value most? [remember, give me an example of good practice… and a bad one]

- When they do NOT do what you expect [those examples of good practice], why do you think they DON’T DO IT?
- Who do you think should evaluate them? Do you think you should be able to evaluate the work of professionals? Why/why not? What do you think of the complaints and satisfaction surveys?
- Finally, do you think it is useful to evaluate the work carried out by nurses? Why/why not? What could it be useful for?

Individual interview: Nursing professionals.

We’re going to talk about what aspects of nursing care should be measured [care techniques, education, treatment, prevention, promotion, support and monitoring].

- [To begin with], do you think all the necessary data is collected? Why not [if applicable]?
- Which aspects of nursing care are currently measured, and which do you think are not measured? Why not [if applicable]?
- [Given “what is not collected” or “why it is not collected”] What do you think should change? Do you have any suggestions regarding… [review points that have come up].
- What do you think could be gained from these changes?
- Finally, what do you think most reflects the work and contribution of the nursing profession to care?

Closure

To recap all the above, ask a final question:

- Of all the above, what is the most important thing to measure in terms of nursing care? [If they respond with several options, request that they rank them in order of priority].

Bring the meeting (focus group or interview) to a close by thanking all the participants for their collaboration and highlighting the contribution of the opinions provided to the development of this study.
